# Comparison of Bifurcated Halogen with Hydrogen Bonds

**DOI:** 10.3390/molecules26020350

**Published:** 2021-01-12

**Authors:** Steve Scheiner

**Affiliations:** Department of Chemistry and Biochemistry, Utah State University, Logan, UT 84322-0300, USA; steve.scheiner@usu.edu; Tel.: +1-435-797-7419

**Keywords:** cooperativity, σ-hole, AIM, NBO, IR, NMR

## Abstract

Bifurcated halogen bonds are constructed with FBr and FI as Lewis acids, paired with NH_3_ and NCH bases. The first type considered places two bases together with a single acid, while the reverse case of two acids sharing a single base constitutes the second type. These bifurcated systems are compared with the analogous H-bonds wherein FH serves as the acid. In most cases, a bifurcated system is energetically inferior to a single linear bond. There is a larger energetic cost to forcing the single σ-hole of an acid to interact with a pair of bases, than the other way around where two acids engage with the lone pair of a single base. In comparison to FBr and FI, the H-bonding FH acid is better able to participate in a bifurcated sharing with two bases. This behavior is traced to the properties of the monomers, in particular the specific shape of the molecular electrostatic potential, the anisotropy of the orbitals of the acid and base that interact directly with one another, and the angular extent of the total electron density of the two molecules.

## 1. Introduction

Linus Pauling’s early explanation [1] of what has come to be called the hydrogen bond (HB) ushered in a long period of its investigation, including follow-up work by himself and coworkers [2,3,4]. Studies on this topic continue to this day, and are summarized periodically by books dedicated to descriptions of developments up to that point in time [5,6,7,8,9,10,11,12,13,14,15,16]. One of the chief advances of recent years has been an expansion of the list of eligible atoms that participate in such bonds [17]. While the early thinking focused on highly electronegative atoms like N, O, and F, this field has been greatly generalized to much less electronegative atoms like C, P, Se, and even metals [18,19,20,21,22,23,24,25,26,27,28,29,30,31,32]. Along with this expansion in terms of atoms, has also come a broader concept of the source of electrons from the nucleophile that extends well beyond a lone pair, to π-systems, σ-bonds, and the half-filled orbitals of radicals [33,34,35,36,37,38,39].

**Scheme 1:**
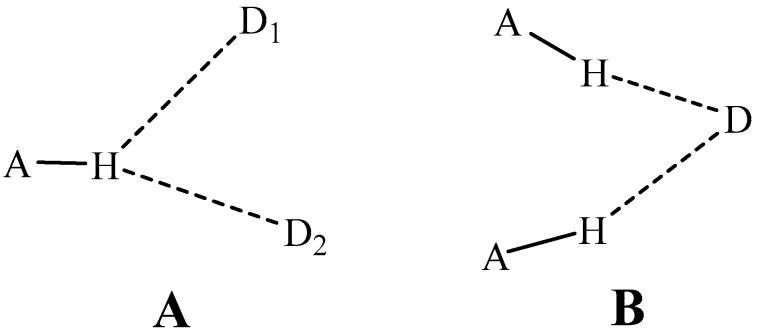
Diagrams of two sorts of bifurcated HBs. (**A**) shares a single proton, and (**B**) shares a single electron donor.

Another extension of the HB concept arises in connection with the number of groups participating. A classical HB places the bridging proton squarely between a proton-donor and acceptor atom, as might occur for example between the two O atoms of the water dimer. While such a geometry might in fact represent a preferred structure, there have been numerous observations of what have come to be termed bifurcated HBs. What is usually meant by this term is the placement of the bridging proton between two different acceptors, D_1_ and D_2_ as depicted in Scheme 1A. Neither of the two alignments in such a proton-shared configuration are linear, and the position of the H is not necessarily precisely symmetric with respect to D_1_ and D_2_. Another sort of bifurcated HB shares a common electron donor between two different proton donors, as indicated in Scheme 1B.

Bifurcated H-bonds of either type are quite common as surveys of crystal structures have revealed [40]. In general, these bifurcated H-bonds have served as fertile ground for study over the years [41,42,43,44,45,46]. The 2-fluoroethanol trimer [47] furnishes one such particular example, where a single H atom can engage with O and N atoms simultaneously [48]. An example drawn from biology [49] occurs within the α-helix N-Caps of ankyrin repeat proteins. Bifurcated H-bonds are also necessary to stabilize fibrils of poly(L-glutamic) acid [50]. Another example [51] places a halide between two H atoms of an alkenic=CH_2_ group.

Due to their similarity to H-bonds it is not surprising that bifurcated halogen bonds are a distinct possibility, and they have undergone some scrutiny as well [52,53,54]. These sorts of bonds are equivalent to those depicted in Scheme 1, but with the H atoms replaced by a halogen atom. There is recent evidence of a bifurcated halogen bond [55] with a C-Br group interacting with both Cl and Pt atoms. In another example, an aryl I atom participated [56] in a bifurcated halogen bond with a pair of O electron donors of a PtO_4_ system. An I atom of a PtI_2_Br_2_ moiety [57] can involve itself in several halogen bonds simultaneously. A halogen atom placed between two N atoms in a bidentate diazaheterocyclic compound [58] provides another example. Even the first-row F halogen appears capable of participating in a bifurcated halogen bond [59] under certain conditions.

Like halogen atoms, chalcogen atoms are also capable of engaging in bifurcated interactions, as for example [60] wherein a single chalcogen atom binds symmetrically to a pair of electron donor atoms. Another example [61] pairs one of the chalcogen (Y) atoms of a Y=C=Y molecule with the two O atoms of 1,2-dihydroxybenzene. Chalcogen bonds can mix with H-bonds in a hybrid sort of bifurcation as found recently in a bifurcated supramolecular synthon in ebselen [62]. Pnicogen atoms are also capable of engaging in bifurcated interactions [63], as can triel atoms, for example when two triels interact with a common halide [64]. The same is true of rare gas atoms [65] which can accept electrons from a symmetrically disposed pair of O atoms.

A great deal has been learned about the halogen bond (XB) in recent years, and its parallels to the HB. Both sorts of bonds involve a strong electrostatic attraction between the electron donor and acceptor groups. While the bridging proton takes on an overall positive partial charge within its A-H subunit, the opposite is true of the X atom. However the electrostatic attraction to the nucleophile is made possible by a small positive region lying along the extension of the A-X bond, commonly referred to as a σ-hole. Both sorts of bonds are stabilized further by charge transfer from the nucleophile into the σ *(A-H/X) antibonding orbital. Also in common, electron-withdrawing substituents on the Lewis acid strengthen either bond by enhancing the positive charge residing on the H or X atom.

While much is now understood about the fundamental properties of standard halogen bonds, there is far less information about the corresponding bifurcated XBs. Under what conditions might they occur, and which or both of types A and B in Scheme 1 are possible? Is there a particular strength or type of base that is required in order for a bifurcated XB to occur? How does the strength of a bifurcated XB compare with that of a single linear bond, and how does bifurcation affect the lengths of the bonds involved? How close together can the pair of bases in Scheme 1A approach one another, and likewise for the two acids in Scheme 1B. Tying all these questions together is a comparison of the properties of bifurcated halogen bonds with the analogous H-bonds.

In order to answer these questions, a number of systems are devised here and evaluated via quantum chemical calculations. FBr and FI are both capable of forming halogen bonds by virtue of fairly deep σ-holes on the Br and I atoms, respectively. The larger size of the I atom imbues it with the ability to form somewhat stronger XBs of the two. FH is employed as the corresponding H-bonding acid for purposes of comparison. Two different bases are considered. NH_3_ is a stronger base than NCH, in part due to its sp^3^ hybridization in comparison to sp for the latter. Each of the three Lewis acids is paired with both NH_3_ and NCH, and for each case both the acid-shared Scheme 1A type and the base-shared type 1B are evaluated.

## 2. Results

**Figure 1:**
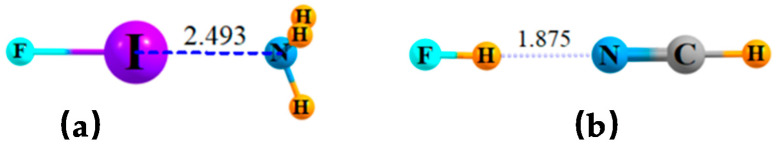
Optimized geometries of (**a**) FI···NH_3_ and (**b**) FH··NCH dyads. Distances in Å.

In the case of simple dimers, whether halogen or H-bonded, the geometric preference is for a linear arrangement, as delineated in Figure 1a,b for sample systems FI··NH_3_ and FH··NCH, respectively. There are two types of bifurcated bonds that might be imagined. In the first place, two separate bases can compete for the same σ-hole on the halogen atom (or the H atom in the case of H-bonds) as delineated in Scheme 1A. An alternate scheme in Scheme 1B would place two Lewis acids in competition for a single lone pair on a base molecule.

### 2.1. Shared Lone Pair of Single Base

**Figure 2:**
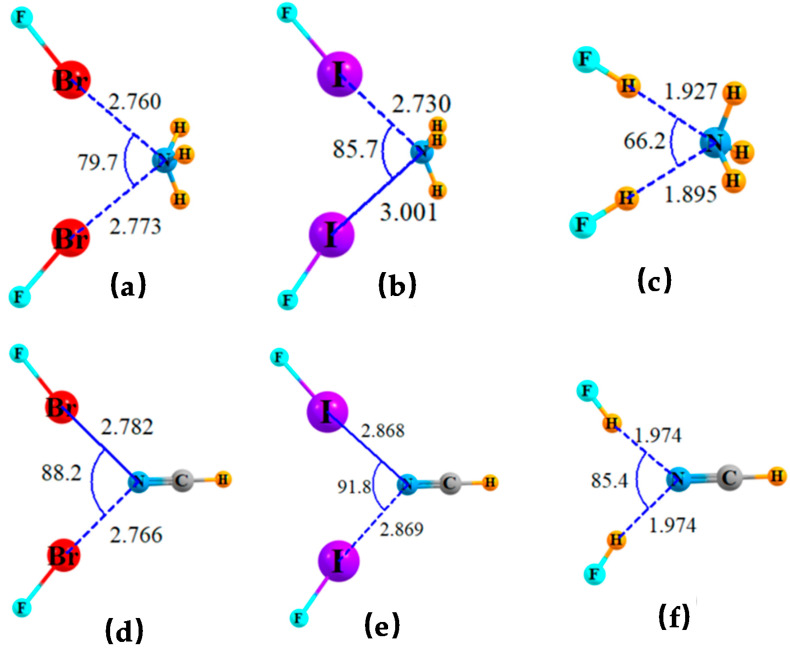
Geometries of triads wherein a pair of Lewis acids share a single lone pair of NH_3_ or NCH, optimized with constraint of equal θ(X··N··X) angles. Distances in Å and angles in degs. NH_3_ is shared by two (**a**) FBr, (**b**) FI and (**c**) FH. NCH is shared by two (**d**) FBr, (**e**) FI, and (**f**) FH.

The systems representing Scheme 1B are illustrated in Figure 2 for both NH_3_ and NCH as bases, and each combined with a pair of FBr, FI, or FH molecules. None of the triads pictured there represent a true minimum. When two acid units were initially placed in positions similar to those in the figure, a full optimization led to large-scale motion of one acid, leaving a single linear dyad as in Figure 1. The remaining acid molecule would completely reposition itself so as to engage in another interaction altogether. For example, one of the two FI molecules would engage in a I··I halogen bond when paired with NH_3_. In the case of the NCH base, the second FI molecule maintained a I··N XB with N of the base, but a highly distorted one. The geometries illustrated in Figure 2 were the result of imposing a restriction that the two acid molecules are symmetrically placed around the base. In the case of 2 FI molecules combined with NCH, for example, both θ(CN··I) angles were forced to be equal to one another. In other words, each of the bifurcated bonding configurations of Figure 2 is less stable than one that contains a single, strong, and linear XB or HB.

A quantitative assessment of the bonding in these triads may be gleaned from Table 1. For each combination of acid and base, the first row provides the parameters for the pure, fully optimized and linear dyads. As an example, the first row shows that the R(Br··N) halogen bond length in FBr··NH_3_ is equal to 2.339 Å. The next row indicates this bond is stretched to 2.760 Å in the triad when a second FBr must share the N lone pair on NH_3_, as in Figure 2a. Note that the triad is not fully symmetric in that the two R(Br··N) distances are not quite equal, so it is the shorter of the two that is catalogued in Table 2. The two Br atoms are situated such that the θ(Br··N··Br) angle is 79.7° in the triad.

The interaction energies in the next column were evaluated by comparing the energy of each complex with that of the sum of monomers which were left in their geometries within the confines of the dimer. These quantities verify the preference of the single XB within the dimer (16.30 kcal/mol) to that of the triad (15.60 kcal/mol) where the two FBr units must share a single N lone pair. The electron density at the Br··N bond critical points in the next column amplify this energetic preference, in that the single linear Br··N density of 0.056 au greatly exceeds the 0.021 au in each of the two bifurcated XBs within the trimer. The same pattern is evident in the NBO values of E(2) for the charge transfer from the N lone pair into the σ *(FBr) antibonding orbitals. This perturbation energy of 51.1 kcal/mol in the linear dimer is much higher than the 9.0 kcal/mol for each of the two XBs in the bifurcated trimer.

It is anticipated that there are two factors contributing to the bond weakening in the triads. In the first place, each bond is distorted from its preferred linear arrangement which can only be achieved within the dimer. The second factor arises since there are two Lewis acids, both competing for the same N lone pair. The fact that the base must serve as electron donor to two acids simultaneously would lead to a negative cooperativity. The third row of each section of Table 1 allows a separation of these two factors. The quantities reported there represent those of a dimer containing only a single acid unit, but it is placed in the same position it occupies within the trimer. In other words, these values document the result of the geometric distortion from the optimized dimer, but absent the negative cooperativity that would arise were the second acid unit physically present.

Again taking the (FBr)_2_NH_3_ system as our example, the placement of a single FBr molecule in the position it occupies in the trimer reduces the interaction energy of the fully optimized dimer (16.30 kcal/mol) down to 9.43 kcal/mol. So, the bifurcation configuration cuts the interaction energy nearly in half. The reduction in ρ_BCP_ is even greater, and E(2) is reduced to only about 1/5 its optimized value. Adding in the second FBr molecule does not double the interaction energy, as would be the case in the absence of any cooperative effects. Instead of 18.86 kcal/mol (twice the value of the individual distorted Br··N), the interaction energy of the trimer is only 15.60, so one might assess the negative cooperativity as their difference, i.e., 3.26 kcal/mol.

Very similar trends can be observed when FBr is replaced by FI. The halogen bonds in the bifurcated triad are stretched relative to the fully optimized linear dimer. The interaction energy within the trimer is less than in the linear dimer, and there is a substantial negative cooperativity. The bifurcated H-bonds associated with a pair of HF molecules are slightly different. There is negligible weakening of the interaction energy on going from dimer to trimer, and the two HF molecules are placed a bit closer together, with θ(H··N··H) only 66°. However, the negative cooperativity remains.

There is a qualitative change when the sp^3^ lone pair of NH_3_ is replaced by the sp-hybridization of NCH. In the first place, the latter is a weaker base, so interaction energies, and their AIM and NBO markers are similarly reduced, and there is a concomitant stretching of the corresponding XBs. However, there is also the change that the interaction energies of the triads are larger than those within the fully optimized linear dimers. The 7.2 kcal/mol interaction energy of the linear FBr··NCH dimer rises to 10.5 kcal/mol when a second FBr molecule is added, and the system is forced into a bifurcated arrangement. In other words, there is an energetic advantage to bifurcation about the NCH lone pair. Part of the reason for this change is that the energetic cost of moving each acid from its fully optimized position to that which it occupies in the bifurcated trimer is less stringent for NCH. For example, the interaction energy of the FBr··NCH dimer is 7.20 kcal/mol, which is reduced only slightly to 6.07 kcal/mol within the context of the trimer structure. The negative cooperativity observed for NH_3_ occurs as well for NCH, so that is a common feature of these bifurcated trimers.

### 2.2. Shared σ-Hole of Single Acid

**Figure 3:**
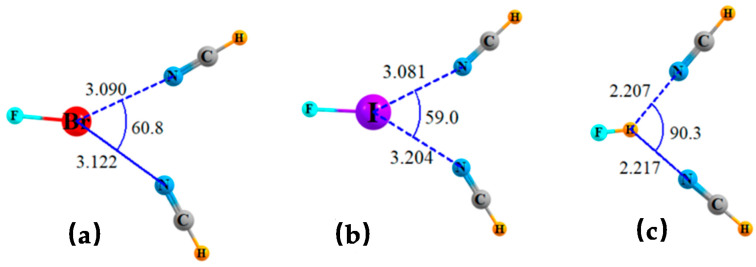
Geometries of triads wherein a pair of NCH bases share a single σ-hole of (**a**) FBr, (**b**) FI, and (**c**) FH, optimized with constraint of equal θ(N··X··N) angles. Distances in Å and angles in degs.

An alternate form of bifurcation would have two separate bases sharing a single σ-hole on a Lewis acid molecule. Systems of this sort are depicted in Figure 3 where two NCH bases are placed accordingly around the FBr, FI, and H-bonding FH molecules. As in the earlier cases, these structures are not the result of a full geometry optimization which aligns one HCN along the FX axis, while the second moves around to bind to the first. The bifurcated structures were derived by enforcing a degree of symmetry wherein the two FX··N angles were restricted to be equal to one another. Bifurcated systems involving NH_3_ rather than NCH were not possible even with such a restriction, since one NH_3_ would rotate so as to engage in NH··N H-bonding with the other.

The geometric and other properties of these bifurcated triads are reported in Table 2, along with the fully optimized dimers containing a linear XB or HB. Unlike the addition of a second FX molecule to the FX··NCH dyads which raised the overall interaction energy, the addition instead of a second NCH base reduces this quantity. Taking FI···NCH as an example, its XB energy is 10.2 kcal/mol. The addition of a second FI raises the total interaction energy of the triad up to 13.5 kcal/mol, whereas this quantity drops down to 6.8 kcal/mol upon addition of a second NCH. Part of the reason for this behavior is the more precipitous drop in the individual interaction energies when the deformation from optimized linear dimer to the bent structure is taken into account. As may be observed in Table 1, this distortion results in FX··NCH interaction energies of some 6–8 kcal/mol, while this quantity drops down to 3–5 kcal/mol in Table 2 when the geometry is influenced by a pair of NCH bases.

This opposite behavior is reflected to some extent in the geometries. The addition of a second Lewis acid lengthens the intermolecular distances by a fairly small amount, between 0.10 and 0.25 Å. These stretches are much longer when it is a second NCH that is added, in the range between 0.33 and 0.46 Å. The latter longer intermolecular distances are a major factor in the reduction of the distorted dimer geometries mentioned above. The pattern continues in the other measures of bonding as well. Both the bond critical point densities and the NBO E(2) quantities are reduced much more by the addition of a second NCH than by a second FX unit. The smaller θ angles in Table 2 as compared to Table 1 show that the two NCH bases can approach one another more closely than the pair of FX molecules. The θ(N··X··N) angles of 59–61° are considerably smaller than the θ(X··N··X) angles of 88–92°. However, there is no such distinction for FH where the corresponding angles are comparable to one another.

### 2.3. Effects of Complexation on Monomers

It is understood that the formation of a HB or XB will affect the internal properties of each subunit. For example, the internal bond length of a FH acid will stretch when engaged in a H-bond, and its stretching frequency shift to the red. These changes will be accompanied by a downfield shift of the bridging proton’s NMR signal, caused in part by its deshielding. The effects of both dimerization and trimerization on the individual monomers are reported in Table 3. The Δr quantities listed there show that complexation results in a stretching of each FX covalent bond. This stretching is accentuated for NH_3_ as compared to NCH, consistent with the stronger basicity of the former. The elongations within the halogen-containing FX are more pronounced than within FH. The degree of stretching is reduced when the fully linear optimized dimer is distorted into the bent bifurcated structures. It might be noted as well that the reductions imposed in the trimers relative to the dyads are smaller when it is a second NCH that is added to FX··NCH, as compared to a second FX.

The next column of Table 3 displays the change in the associated ν(FX) stretching frequency, which is a negative red shift in the majority of cases. These shifts are particularly pronounced for FH with the much lighter H as compared to the halogenated FX units. Like the FX elongations, the shifts are larger for NH_3_ as compared to NCH. Also consistent with bond length changes, there is less of an effect arising from addition of a second NCH to the dimer than occurs from addition of FX.

The last three columns of Table 3 contain the changes in chemical shielding arising on the X, F, and N atoms that accompany the formation of the indicated dyads and triads. The shieldings of the halogen atoms are increased by the complexation, particularly Br, while H is deshielded. The F atom is deshielded, as is the basic N atom. In the majority of cases, these NMR properties share the common behavior that the change in shielding occurring within the dimer is attenuated within the trimer. With respect to the Lewis acid X and F atoms, one sees again the pattern noted earlier for the energetics, that the addition of a second NCH unit has a greater effect than does a second FX.

### 2.4. Understanding the Trends

The data show that enforcing a symmetric sort of bifurcated pair of bonds is energetically inferior to a single linear such bond in most cases. The exceptions arise when a pair of H or X bonding acids interact with a single weak NCH base, wherein the total interaction energy of the triad is slightly larger than that of a single linear bond. A refined examination of the numerical data indicates that there is less of an energetic cost when two acids share a single lone pair, than the reverse situation wherein a single acid σ-hole must accommodate two bases. It is not only the energy itself, but the other indicators of noncovalent bond strength, AIM, and NBO markers, as well as geometric and spectroscopic data, that affirm the angular deformations necessary to align two bases with a single σ-hole is more damaging than when two acids share a single base lone pair.

**Figure 4:**
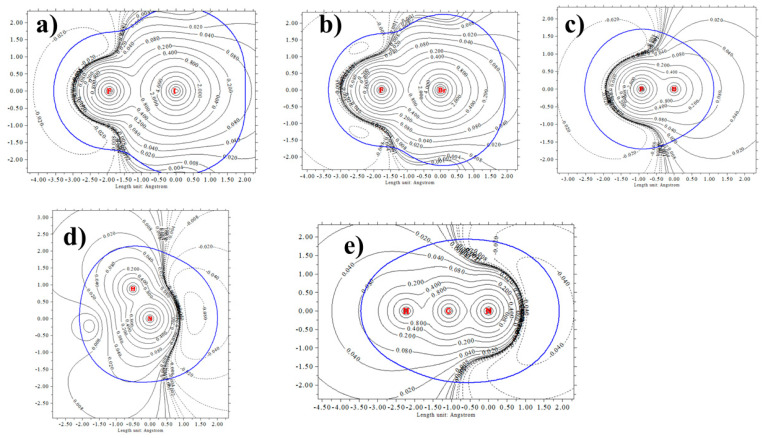
Contour plots of MEP of (**a**) FI, (**b**) FBr, (**c**) FH, (**d**) NH_3_ and (**e**) NCH. Blue line represents a vdW surface defined as isodensity ρ = 0.001 au.

It is possible to understand the underlying reasons for this distinction by examination of the molecular electrostatic potential (MEP) that surrounds each molecule. Figure 4a–c presents the MEP of each of the three Lewis acid molecules in the form of a contour plot. The blue curve indicates the van der Waals surface of each, as measured by an isodensity surface with ρ = 0.001 au. Focusing attention on the right side of each which encompasses its σ-hole, one can see that this vdW surface crosses several different contours, an indication of its anisotropy. In other words, the MEP drops in value quickly as the reference point moves away from the F-X axis. This anisotropy appears to be more dramatic for FBr and HI than for FH in Figure 4c. This behavior can be contrasted with the N lone pair region of the NH_3_ and NCH bases, whose negative MEP with its excess density might be termed a σ-lump. Their MEPs, displayed as the broken contours on the right sides of Figure 4d,e, respectively, undergo less crossing of the blue vdW surface, particularly for NCH.

**Figure 5:**
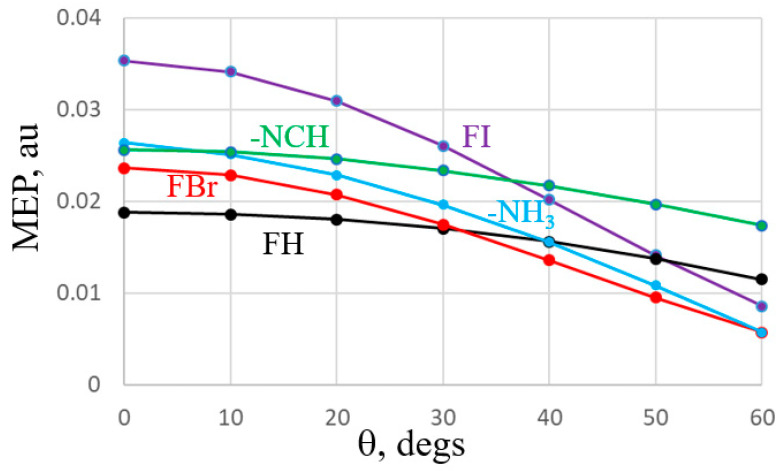
MEP of indicated molecule as function of θ defined as deviation from FX axis for acids, NCH axis for NCH, and C_3_ symmetry axis of NH_3_. Potentials were evaluated along a circle centered about the X or N atom, with a radius of 3 Å. The negative of the potential is shown for NCH and NH_3_.

An alternate manner of viewing the comparative anisotropies is via Figure 5 which plots the MEP on a circle of constant distance from the Br, I, and H atoms of the acids, and the N atom of the two bases. (Note that the sign of the MEP has been reversed for the two bases for purposes of comparison with the acids.) The red (FBr) and purple (FI) curves display the greatest drop as θ increases away from the F-X axis, while the black FH curve is a bit flatter. The blue curve associated with the anisotropy of the σ-lump of NH_3_ is much steeper than that for NCH.

These visualizations conform with the numerical data. The greater anisotropy of the NH_3_ σ-lump region as compared to NCH accounts for the observation that the angular deformations required to add two acids is more energetically costly for the former. Moreover, the high anisotropy of the FBr and FI σ-holes explains the large drop in interaction energy when two bases are placed around them that are forced to lie off of the F-X axis.

**Figure 6:**
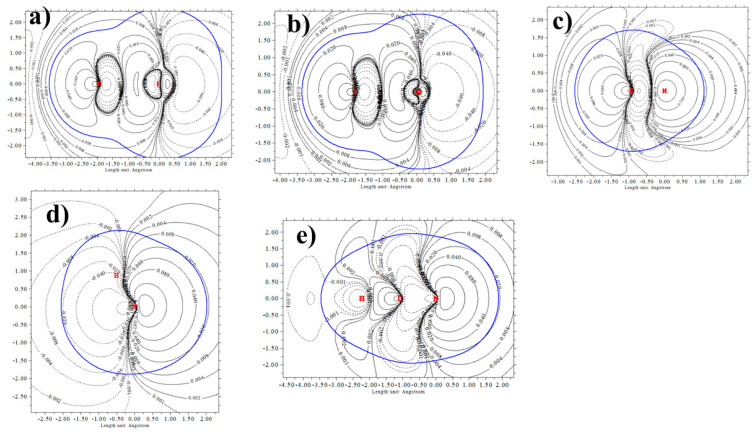
Contour plots of the σ *(FX) antibonding orbital (**a**) FI, (**b**) FBr, (**c**) FH, and the N lone pair orbitals of (**d**) NH_3_ and (**e**) NCH. Blue line represents a vdW surface defined as isodensity ρ = 0.001 au.

Of course, the electrostatic interaction represents only one chapter of the story. As represented through the NBO formalism, the bonding is partly due also to charge transfer from the N lone pair orbital to the unoccupied σ *(FX) orbital of the acid. This component too ought to have an angular dependence that diminishes as the bond is forced from linearity. The angular characteristics of the relevant NBO orbitals are provided in Figure 6 in the form of contour plots, where the blue curve again indicates the vdW surface of each unit. The diagrams are not unlike the MEPs of Figure 4 in that the σ * orbitals of FBr and FI cross the most contour lines. The σ *(FH) orbital is a bit more isotropic. Most isotropic of all are the N lone pair orbitals in Figure 6d,e where the blue vdW surface nearly traces out a single contour line for much of its path.

**Figure 7:**
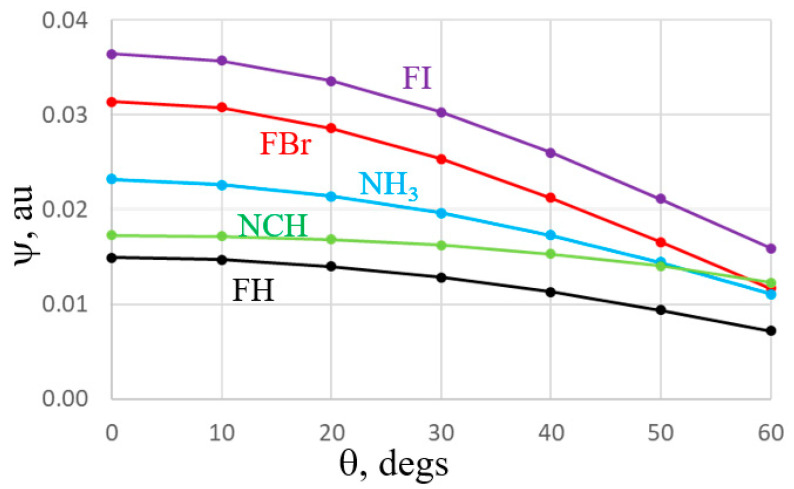
Amplitudes of the σ *(FX) antibonding orbital of FI, FBr, FH, and the N lone pair orbitals of NH_3_ and NC, plotted against deviation angle θ from FX axis for acids, NCH axis for NCH, and C_3_ symmetry axis of NH_3_. Wave functions were evaluated along a circle centered about the X or N atom, with a radius of 2 Å for all except 1.5 Å for FH.

The near isotropy of the NCH lone pair is particularly obvious by the nearly flat nature of the green curve in Figure 7. The blue NH_3_ curve is a bit more anisotropic, reminiscent of the MEP in Figure 5. Also in common with the MEPs, it is again the FBr and FI curves which diminish the most as one shifts further from the F-X axis. So, both the MEP and individual orbital diagrams demonstrate that much of the behavior of the bifurcated systems is consistent with both an electrostatic or orbital overlap interpretation.

The anisotropies of the MEP and lone pair orbital of the bases fit squarely with the geometries of the bifurcated systems in Figure 2. In all cases, whether the two acids are FBr, FI, or FH, these acids are situated further apart from one another when paired with NCH than with NH_3_. Figure 5 and Figure 7 are consistent with this wider spacing as the MEP and lone pair orbital around the N atom both deteriorate more slowly as one shifts away from the linear arrangement of NCH vs. NH_3_, so can tolerate a wider displacement, which in turn minimizes repulsions between the two acids. The same can be said for the structures exhibited in Figure 3 where two bases are paired with a single acid. The two bases are spaced most closely together for FI, which also has the quickest deterioration of the σ-hole and σ * orbital. These two properties diminish much more slowly for FH, which allows 30° greater separation of the two bases in Figure 3c, again minimizing base–base repulsions.

**Figure 8:**
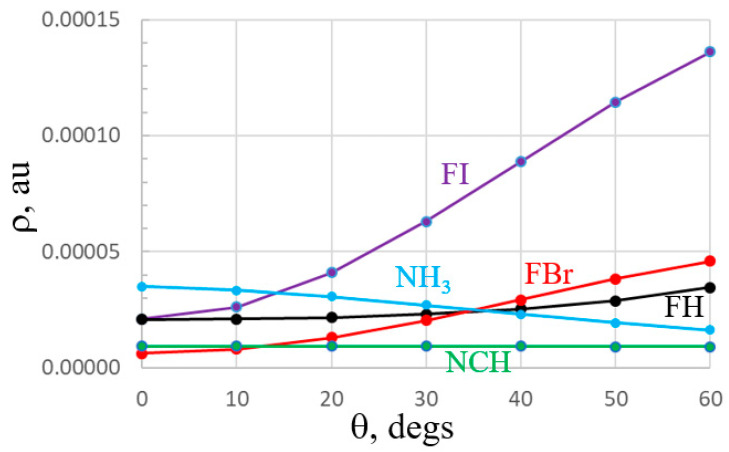
Total electron densities of indicated monomers, plotted against deviation angle θ from FX axis for acids, NCH axis for NCH, and C_3_ symmetry axis of NH_3_. Potentials were evaluated along a circle centered about the X or N atom, with a radius of 3 Å for all except 2 Å for FH.

Still another factor in the ability of a partner molecule to locate itself off of the primary axis is the anisotropy of the electron density. A high electron density would tend to increase the exchange repulsion with its partner. An example may be drawn from the purple FI plot of the electron density around FI, at a constant distance of 3 Å from the I atom in Figure 8. This density rises rather dramatically with θ which would impede a base from shifting off of the FI axis, thus hindering the ability of FI to engage with two bases, each of which would have to move in this direction. The minimum in the density along the FI axis is consistent with the basic idea of a depleted σ-hole that sucks density out of this region and the concept of polar flattening. A similar rise, albeit not quite as steep, is observed in the red FBr curve, and the increase is even more gradual for FH. The bases behave quite differently. The change in ρ as θ increases is either barely perceptible as in the case of NCH, or even a decrease for NH_3_. This maximum at θ = 0 comports with the idea of a σ-lump that aligns with the N lone pair. The absence of a higher electron density resulting from a displacement off the lone pair direction is another factor accounting for the greater ability of the bases to share a lone pair with two Lewis acids, as compared to the diminished capability of a Lewis acid to share a single σ-hole with two bases. This same feature also helps explain the greater separation of the two FX acids in Figure 2a,b (~90°) as compared to the 60° separation of the two bases in Figure 3a,b.

**Figure 9:**
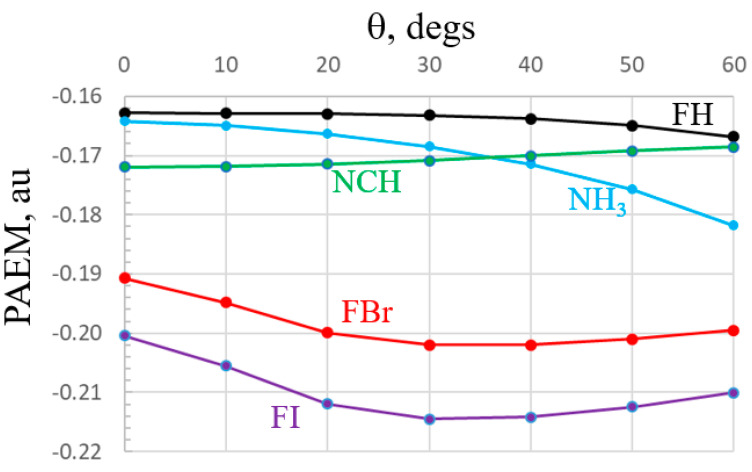
PAEM of indicated monomers, plotted against deviation angle θ from FX axis for acids, NCH axis for NCH, and C_3_ symmetry axis of NH_3_. Quantities were evaluated along a circle centered about the X or N atom, with a radius of 3 Å.

With regard to the electrostatic potential surrounding each monomer, an alternate view of this quantity is known as the potential acting on one electron in a molecule (PAEM) [66] which contains exchange as well as Coulomb elements. This quantity has seen some applications in systems containing halogen and other sorts of noncovalent bonds [67,68]. The PAEMs of the five monomers are displayed in Figure 9 as a function of the displacement from their symmetry line, all with constant distance of 3 Å from the central atom. It might be noted first that unlike the MEP which is positive in the σ-hole regions of the Lewis acids, and negative along the lone pairs, the PAEM is negative for all. Like the MEP, the PAEM of FI and FBr have maxima along the F-X axis, but they do not decline monotonously, instead reaching minima at about 30°. The PAEM of FH is very flat until finally tailing off near 60°. Bases NCH and NH_3_ behave oppositely from one another. While the latter has its least negative value along the lone pair direction, this quantity is most negative along the lone pair of NCH.

## 3. Discussion

According to the calculations discussed above, a bifurcated arrangement which encompasses a pair of distorted noncovalent bonds is less favorable than is a single, fully optimized linear arrangement even though the former has two such bonds to the single bond in the latter. This situation applies to H-bonds as well as halogen bonds. In fact, a geometry encompassing a bifurcated pair of noncovalent bonds to either a common lone pair or a common σ-hole does not represent a true minimum on the potential energy surface of these triads. The exceptions occur when a pair of H or X bonding acids interact with a single weak NCH base, wherein the total interaction energy of the triad is larger than that of a single linear bond, even if only by a small amount.

This situation arises due to several principal factors. Angular deformations implicit to bifurcated bonds cause a significant weakening. Coupled to this issue is the negative cooperativity that arises when a single molecule acts as either double electron donor or acceptor. There are also repulsive interactions between the pair of molecules competing to bond with the central unit. These repulsions are less than 2 kcal/mol for a pair of FBr or FI units but are a bit larger for two FH or NCH molecules. Were these repulsions to be removed from the triad interaction energies, the remaining quantity representing only the attractions between the central NH_3_ and the two FX acids is no longer less than that within a fully optimized linear FX··NH_3_ dimer. However, even removal of the repulsion between the two NCH bases in the FX(NCH)_2_ triads does not raise the remaining pair of FX··N attractions up to the level of a single linear FX··NCH dimer, underscoring the lesser ability of a σ-hole to accommodate two separate bases.

All energetics have been corrected for basis set superposition error by the counterpoise protocol. Failure to make this correction enlarges each of the interaction energies in Table 1 and Table 2 by a small amount, roughly 1 kcal/mol. Moreover, the uncorrected energies obey the same trends as the properly corrected values. As a second point, the interaction energies which are the focus here refer to individual monomers in the geometries they adopt within the complex. If instead, the referrals are to the monomers in their fully optimized geometries, the resulting binding energies differ very little from the interaction energies, by less than 1 kcal/mol. So, again the trends displayed in the tables here would be unchanged if binding energies were considered instead.

Although there is an innate energetic preference in many cases for a single noncovalent XB or HB as opposed to a bifurcated arrangement, the latter obviously does arise rather commonly within the confines of larger macromolecular systems, or when crystal packing forces are present. Configurations found there reflect the fact that external forces preclude the optimal linear arrangement. In such a situation, it makes sense that a second Lewis acid can take advantage of the unoccupied side of the base’s lone pair, which is preferable to the former lying dormant. After all, a bent bond is preferable to no bond at all. The data in Table 1 and Table 2 support this contention, in that there is a strong energetic advantage in adding a second bond to an already bent bond of the same type. Taking the XB formed between FBr and NH_3_ as an example, the interaction energy of a bent XB is 9.4 kcal/mol, which grows to 15.6 kcal/mol upon formation of a second FBr··N XB.

The bifurcated bonds considered here are idealized in the sense that they are symmetric. That is, the two CN··X angles when a pair of FX acids interact with the NCH lone pair were held to be equal to one another, as was true for the NH_3_ base, as presented in Figure 2. Likewise, the two FX··N angles in Figure 3 were restricted to be equivalent. This idealized situation obviously does not represent the general bifurcated case where there is no restriction to a symmetric structure. What the work presented here has demonstrated is that there will be a natural tendency for one of the two bonds to lean toward linearity, slowly squeezing the other bond out of its way if conditions permit. However, if this approach toward linearity is not precluded by circumstances beyond its control, a bifurcated arrangement comprising two noncovalent bonds would be the second-best scenario.

The work described here considers the case where the nucleophile contains only a single lone pair that must be shared between two Lewis acids. However, when this restriction is lifted, allotting more than one lone pair to the base, it becomes much easier for the latter to participate in multiple noncovalent bonds simultaneously. Recent examples can be drawn from the rapidly developing field of selective anion binding. The base in question is typically a halide anion containing not only several available lone pairs, but also a full negative charge. Bulfield and Huber [69] have reviewed the manner in which catalysis may be facilitated by the bifurcated halogen bonding of an anion to a bipodal receptor with two symmetrically disposed halogen atoms. Lim et al. [70] have extended these same ideas to chalcogen bonds, where again an anion is bound by a bifurcated pair of noncovalent bonds to a bipodal receptor. Quantum calculations have explored this sort of bonding, and extended the ideas to bifurcated pnicogen, chalcogen, and tetrel bonds as well [71,72]. Other studies have extended these ideas to more than two noncovalent bonds to a given anion, again containing multiple lone pairs, in a general sense where the two electron-accepting atoms are located on different molecules [73,74,75].

There is precedent for the finding that is confirmed here that the halogen bond tends more toward linearity than does the H-bond [76]. For purposes of interpretation, the calculations presented here have focused on the directionality of the MEP and the electron density, expressed as either its total or individual MOs, to understand the ability of different sorts of bonds to accommodate bifurcated interactions. Indeed, there is precedent in the literature for the idea that both electrostatics and charge transfer/induction are important components of the anisotropy of noncovalent bonds [77,78,79,80,81,82]. There is likewise accumulating evidence that electron repulsion also plays an important role [77,78,83,84,85,86,87,88].

The work presented here has focused on halogen bonds, and their potential to share their single σ-hole with a pair of nucleophiles. Specifically, the halogen atoms considered here are all univalent in the sense that they are involved in only one covalent bond, in this case to F. However, halogen atoms can participate in hypervalent situations where they may be involved in more than one covalent bond. In such a situation one can anticipate that there will be one σ-hole that corresponds to each such internal bond. Kirshenboim and Kozuch [89] explored this scenario, and the manner in which the multiple σ-holes might affect the halogen bonding. Heinen et al. [90] later documented these ideas in the particular case of the hypervalent I atom through both experimental and computational means. The ability of a single Lewis acid atom with multiple σ-holes to engage in several noncovalent bonds has been explored also in the framework of chalcogen, pnicogen, and tetrel bonds [91,92,93,94,95,96,97]. These sorts of hypervalent bonding situations, with multiple σ-holes, are thus much more amenable to bifurcated arrangements than is the univalent scenario.

## 4. Computational Methods

All quantum calculations were carried out within the framework of the Gaussian-09 set of codes [98]. DFT procedures were used to include electron correlation via the M06-2X functional along with the aug-cc-pVDZ basis set. Relativistic effects which may be important for the heavy I atom were incorporated through the aug-cc-pVDZ-PP pseudopotential [99,100]. There exists ample evidence of the ability of this level of theory to accurately reproduce results from much higher levels when applied to noncovalent interactions of this type [30,101,102,103,104,105,106,107,108,109]. The interaction energy, E_int_, of each complex was computed as the difference between the energy of the complex and the sum of the energies of monomers in the geometries they adopt within the complex. Basis set superposition error was corrected via the standard counterpoise [110,111,112] protocol. Molecular electrostatic potentials (MEPs) were computed in the framework of the Multiwfn program [113]. The topology of the electron density was analyzed with the aid of AIM procedures, applying the AIMALL program [114]. The natural bond orbital (NBO) prescription [115,116] was used to elucidate charge transfers and associated energetics between individual orbitals.

## 5. Conclusions

With some exceptions, a bifurcated pair of either H-bonds or halogen bonds is energetically inferior to a single such bond within a dimer. There is a larger energetic cost to forcing the single σ-hole of an acid to interact with a pair of bases, than the other way around where two acids engage with the lone pair of a single base. This distinction can be traced to details of the MEP that surrounds the various molecules, as well as to the shape of the pertinent molecular orbitals, and the total electron density. The acid’s σ-hole is more anisotropic than is the negative MEP at the site of the base’s lone pair. The same is true of the σ *(FX) antibonding orbital which is more directional than is the N lone pair orbital. On both of these scores, the weaker NCH base is less anisotropic than is NH_3_, which allows the former to better accommodate a bifurcated arrangement with two acid molecules. Likewise, the lesser anisotropy of FH as compared to FBr and FI allows H-bonds to better share a single proton than can XBs share their X atom. A shift of a base off of the bond axis of the Lewis acid would encounter a higher electron density, and associated exchange repulsion, another factor inhibiting the formation of a bifurcated bond with two bases, particularly for FI. In contrast, there is no such exchange repulsion factor inhibiting the sort of nonlinear geometry required to link two acids to a single base.

## Figures and Tables

**Table 1:** Intermolecular distances (R, Å), interaction energies (kcal/mol), bond critical point density (ρ_BCP_, 10^−4^ au), and NBO N_lp_→σ *(FX) perturbation energy E(2) (kcal/mol) for complexes where one or two Lewis acids share a single lone pair on the N-base, and θ(X··N··X) angle (degs) for bifurcated bonds.

Base	Acid	R	θ	-E_int_	ρ_BCP_	E(2)
NH_3_	1 FBr	2.339		16.30	560	51.10
	2 FBr	2.760	79.7	15.60	207	8.95
	1 FBr(def) ^a^			9.43	213	10.71
NH_3_	1 FI	2.493		19.17	465	43.61
	2 FI	2.730	85.7	17.51	263	14.65
	1 FI(def)			11.63	267	16.37
NH_3_	1 FH	1.672		13.88	534	40.09
	2 FH	1.895	66.2	13.83	290	13.85
	1 FH(def)			9.73	290	15.44
NCH	1 FBr	2.629		7.20	247	12.13
	2 FBr	2.766	88.2	10.51	178	5.68
	1 FBr(def)			6.07	179	6.33
NCH	1 FI	2.621		10.20	301	20.09
	2 FI	2.868	91.8	13.48	173	6.66
	1 FI(def)			8.05	175	7.73
NCH	1 FH	1.875		7.32	271	12.29
	2 FH	1.974	85.4	9.37	205	5.89
	1 FH(def)			6.04	205	6.51

^a^ single acid unit in geometry adopted within triad.

**Table 2:** Intermolecular distances (R, Å), interaction energies (kcal/mol), bond critical point density (ρ_BCP_, 10^−4^ au), and NBO N_lp_→σ *(FX) perturbation energy E(2) (kcal/mol) for complexes where one or two NCH bases share a single σ-hole on the acid, and θ(N··X··N) angle (degs) for bifurcated bonds.

Acid	Base	R	θ	-E_int_	ρ_BCP_	E(2)
FBr	1 NCH	2.629		7.20	247	12.13
	2 NCH	3.090	60.8	4.31	111	1.86
	1 NCH (def) ^a^			3.33	112	1.93
FI	1 NCH	2.621		10.20	301	20.09
	2 NCH	3.081	59.0	6.83	139	3.74
	1 NCH (def)			5.17	139	3.96
FH	1 NCH	1.875		7.32	271	12.29
	2 NCH	2.207	90.3	6.46	144	2.87
	1 NCH (def)			4.66	147	2.32

^a^ single base unit in geometry adopted within triad.

**Table 3:** Changes relative to monomer of internal bond length (Å), stretching frequency (cm^−1^), and NMR chemical shieldings (ppm) in dyads and triads ^a^.

Base	Acid	Δr	Δν(FX)	Δσ(X)	Δσ(F)	Δσ(N)
NH_3_	1 FBr	0.061	−107.2	1670.5	−790.8	−17.6
	2 FBr	0.012	−27.0	687.5	−168.0	−4.3
NH_3_	1 FI	0.055	−48.5	71.5	−421.3	−7.0
	2 FI	0.013	+8.1	37.7	−214.1	+10.3
NH_3_	1 FH	0.036	−884.8	−7.4	−35.9	−8.8
	2 FH	0.010	−275.6	−3.0	−21.7	−10.6
NCH	1 FBr	0.013	−29.1	680.6	−183.8	−21.6
	2 FBr	0.006	−8.7	484.5	−122.0	−12.8
NCH	1 FI	0.026	−26.2	48.8	−297.0	−12.7
	2 FI	0.008	+15.4	30.4	−173.1	−2.6
NCH	1 FH	0.008	−235.5	−2.2	−6.9	−19.4
	2 FH	0.005	−130.1	−1.6	−11.9	−21.6
1 NCH	FBr	0.013	−29.1	680.6	−183.8	−21.6
2NCH		0.013	−20.5	78.2	−66.5	−45.2
1 NCH	FI	0.026	−26.2	48.8	−297.0	−12.7
2NCH		0.020	−20.7	16.3	−136.1	−41.1
1 NCH	FH	0.008	−235.5	−2.2	−6.9	−19.4
2NCH		0.009	−182.7	−1.3	−16.3	−41.4

^a^ average of two values when there are two equivalent properties.

## Data Availability

The original data presented in this study are available by request to the author.

## Sample Availability

Not available.
